# Cross-Cultural Adaptation of the Tailored Activity Program for Brazilian Portuguese

**DOI:** 10.1093/geroni/igaa057.2160

**Published:** 2020-12-16

**Authors:** Marcia Novielli

**Affiliations:** University of Sao Paulo, Santos, Sao Paulo, Brazil

## Abstract

Brazil lacks an Occupational Therapy methodology of action, justifying the cross-cultural adaptation of TAP. Objectives were to adapt TAP reference materials to the Brazilian culture and evaluation of the applicability of the Portuguese version by perceptions of Occupational Therapists (OT) and family caregivers. The methodology used translation, back translation, evaluation of semantic, idiomatic, conceptual and cultural equivalences and pre-test of materials for production in Portuguese. The OT applied the translated version and evaluated its applicability. Caregivers evaluated the social impact of the adapted program. The cross-cultural adaptation process adapted the entire materials program to Portuguese culture. The OT perception is a need to include one session to guide caregivers and to modify the cognitive assessment used. The caregivers pointed out that TAP helps them in understanding and caring for the elderly with dementia. The TAP-BR has been adapted to the Brazilian culture. Part of a symposium sponsored by the Behavioral Interventions for Older Adults Interest Group.

